# The Effect of Partnership Care Model on Mental Health of Patients with Thalassemia Major

**DOI:** 10.1155/2017/3685402

**Published:** 2017-06-21

**Authors:** Afzal Shamsi, Fardin Amiri, Abbas Ebadi, Musab Ghaderi

**Affiliations:** ^1^Department of Medical-Surgical Nursing, School of Nursing and Midwifery, Tehran University of Medical Sciences, Tehran, Iran; ^2^Department of Community Health Nursing, School of Nursing and Midwifery, Tehran University of Medical Sciences, Tehran, Iran; ^3^Behavioral Sciences Research Center, Nursing Faculty, Baqiyatallah University of Medical Sciences, Tehran, Iran; ^4^Research Center, Jiroft University of Medical Sciences, Jiroft, Iran

## Abstract

**Background:**

Thalassemia major has become a public health problem worldwide, particularly in developing and poor countries, while the role of educating the family and community has not been considered enough in patients' care.

**Objectives:**

This study examines the impact of partnership care model on mental health of patients with beta-thalassemia major.

**Materials and Methods:**

This experimental study, with pretest and posttest design, was performed on patients with beta-thalassemia major in Jiroft city. 82 patients with beta-thalassemia major were allocated randomly into two groups of intervention (41 patients) and control (*n* = 41) groups. Mental health of the participants was measured using the standard questionnaire GHQ-28 before and after intervention in both groups. The intervention was applied to the intervention group for 6 months, based on the partnership care model.

**Results:**

There were significant differences between the scores of mental health and its subscales between two groups after the intervention (*P* < 0.05).

**Conclusions:**

The findings of the study revealed the efficacy and usefulness of partnership care model on mental health of patients with beta-thalassemia major; thus, implementation of this model is suggested for the improvement of mental health of patients with beta-thalassemia major.

## 1. Introduction

Thalassemia is a public health problem worldwide, particularly in developing and poor countries [1]. According to the World Health Organization report, more than 15 million people suffer from thalassemia worldwide [2] and annually about 56 thousand children are born with it in the world [3]. Iran is placed on the thalassemia belt in the world and about 1–10% (average 5.4%) of people are carriers of the disease [4]. There are now about 3 million people carrying the defective gene in Iran and about 25 thousand patients with thalassemia major are identified [5]. With these numbers, Iran ranks first in the world regarding the ratio of patients with thalassemia to the total population [6]. This disease is a major problem, not only for patients and their families, but also for public health system in any country, considering the care and treatment costs, including regular infusions of Deferoxamine Mesylate (desferal), recurrent hospitalization, and other medical procedures [7, 8]. Currently, the treatment of thalassemia major in Iran is through blood transfusion and using intravenous (Deferoxamine Mesylate), oral (Deferasirox), or combined chelators therapy [9]. The cost of treatment is covered by insurers. The goal of treatment is to maintain hemoglobin levels at at least 10 g/dL in both genders. The blood volume for transfusion is calculated based on the patient's hemoglobin (10–15 mL/kg). Treatment with chelators starts after the first 10–20 blood transfusions or when blood Ferritin level reaches more than 1000 ng/mL [10]. Intravenous chelators are routinely used in chelation therapy. Oral or combined chelator's therapy is used when the patient is unable to tolerate intravenous chelators or when sufficient intravenous chelators are not available. Deferoxamine is injected subcutaneously using an injection pump at a rate of 20–60 mg/kg of body weight usually over 8- to 12-hour period, and 3 to 5 times a week [11].

Complex medical care and lifelong unpleasant clinical self-management regimen have adverse effects on mental function and mood of patients and their families [12, 13]. Studies have shown that these patients suffer from psychological problems, such as anxiety and depression, and may be easily damaged by these problems [12]. Another important point is that this disease affects the patients' health and causes physical disorder, growth retardation, and late puberty [14] which affects their self-conscious and will ultimately increase the patient's anxiety and negatively affect their lives [15].

In developing countries, adults with thalassemia major are not treated because of lack of public awareness and unavailability of drugs and experience mood disorders, including despair, sadness, hostility, depression, anxiety, fear of death, lack of self-esteem, isolation, and anger [7, 16]. On the other hand, complications of this disease increase with age and make the patient more tired [17]. This issue will disrupt the self-care and psychomental status and interrupt the treatment procedure [18]. Nurses are on the front lines of providing care to the patients and witnessing the patients' problems. They need to know about the patient's psychomental status in order to provide the most appropriate care plan. In addition, the knowledge about these kinds of problems can help nurses to improve the quality of care in ways that enhance quality of life in patients. Endemic care protocols can be an appropriate guide for improvement of care; thus, this study aimed to use an endemic model, name partnership care model for patients with thalassemia major, and evaluate its effect on mental health (depression and anxiety) of these patients. Partnership care model was first developed, implemented, and evaluated by Alijany-Renany et al. [19]. In this model, the theory of collaboration in the care has been processed. In this model, in the care process, the quality and type of communication between the two sides of the relationship are crucial. In this regard, knowledge, skill, and special tools for treatment and care are the next important issues. Originality and effectiveness of care depend on the correct and favorable formation of nature and quality of care. Therefore, the partnership care model is a regular process for efficient, interactive, and persistent communication between the patient and the nurse to identify the needs and problems and sensitizing the patients to accept continuous health behaviors and help improve their health. The objectives of this model include (1) to establish an efficient, interactive, and persistent relationship between team members in the process of care and treatment, (2) to increase the cooperation, team motivation, and accountability in the process of care and treatment, (3) to increase satisfaction and quality of life of patients, and (4) to reduce complications and risk factors. To achieve the purposes of the model, the designed steps have been arranged in a regular structure, which acts as interconnected and dynamic series, through observing the relationship and logical and evolutionary sequence. These steps include (1) motivation, (2) readiness, (3) involvement, and (4) evaluation, which is predicted for each specific action program [19, 20]. This study investigates the effect of this model on the mental health of patients with thalassemia major. We hypothesized that the partnership care model could improve both the overall health and quality of life of patients.

## 2. Methods

### 2.1. Design and Participants

**Figure 1:**
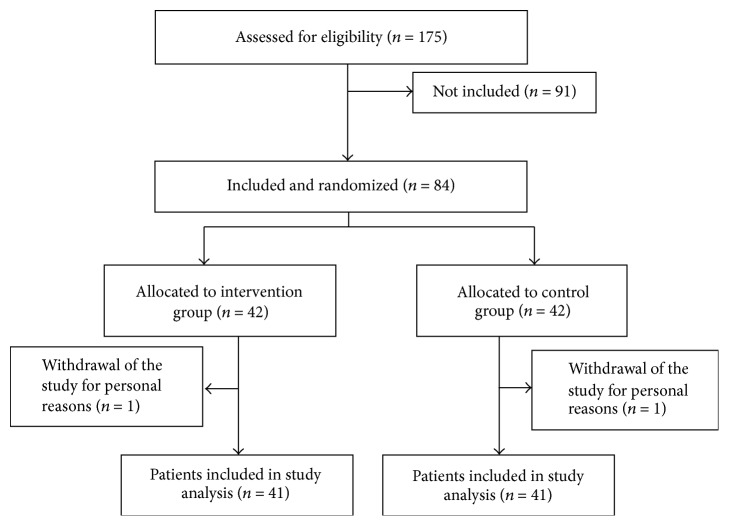
Flowchart of sampling.

This experimental study with pretest and posttest design was conducted in Jiroft, Kerman Province, Iran, on patients with beta-thalassemia major. The study population included patients with beta-thalassemia major (*n* = 175) who referred to the Thalassemia Center of Jiroft city, which included 82 patients of the study sample. The samples were allocated randomly into two groups of intervention (41 patients) and control (*n* = 41) groups (Figure 1). Partnership care model (motivation, readiness, involvement, and evaluation) was conducted for 6 months for the intervention group. Inclusion criteria for this study consisted of patients with thalassemia major, receiving intravenous chelators (Deferoxamine), with the ability to communicate and give information and no history of psychiatric illness.

### 2.2. Procedures

This study was approved by the ethics committee of the Jiroft University of Medical Sciences (permit number: D-93-18) and conforms to the tenets of the declaration of Helsinki. All patients entered the study voluntarily, with consent, and without paying any fee. Informed written consent was obtained from all participants before data collection. At the time of the study, there were no missing data in the samples. Partnership care model was implemented in the intervention group (Table 1). Participants in the control group received no intervention. The data were collected using demographic information questionnaires and standardized General Health Questionnaire (GHQ-28) from both the intervention and the control groups in two steps (before and after intervention). The questionnaire was completed by one of the nursing staff (researcher member) of Thalassemia Treatment Center through interviewing the patients.

In the demographic questionnaire, variables such as age, sex, marital status, education level, age at diagnosis, history of other diseases, and family status were evaluated. By the GHQ-28 questionnaire, data related to health (including mental health) were collected.

### 2.3. Instruments

The data gathered in this study included demographic questionnaire and the standardized questionnaire GHQ-28. Demographic questionnaire was developed by researchers based on scientific resources and on the objectives of the study. The questionnaire included questions about age, gender, marital status, education, family status, age at diagnosis of thalassemia, and history of other diseases. Content validity was determined using comments of 10 relative nursing faculty members and four blood specialists and psychiatrists. To determine reliability, test-retest was used. The questionnaire was completed by thirty patients with thalassemia and repeated after two weeks. The reliability of the tool was approved with an alpha of 88%.

GHQ-28 questionnaire was designed in 1979 by Goldberg and Hiller to screen nonpsychopathic psychological disorders and includes four subscales: physical complaints, symptoms of anxiety, social dysfunction, and depressive symptoms. Each subscale consists of 7 statements and each statement is scored on a Likert scale of 0–3 points and higher scores indicate poorer mental health. In each subscale, scores over 6 and total scores above 22 indicate disease. Goldberg calculated and confirmed Cronbach's alpha coefficient of 0.89 for this tool [21] and the test-retest, split-half reliability, and Cronbach's alpha coefficients were 0.70, 0.93, and 0.90, respectively, and concurrent validity of the questionnaire was determined at 0.55 [22]. Researchers have introduced the GHQ-28 tool as the best tool in the age group 12–18 years [23, 24].

### 2.4. Data Analysis

Data was analyzed using SPSS 20 software, paired *t*-test, independent *t*-test, and chi-square analysis. *P* < 0.05 was considered as statistically significant.

## 3. Results

Mean age of patients in the intervention group was 15.25 ± 3.17 and in the control group was 15.03 ± 3.36 (*P* > 0.05). There was no significant difference between the two groups regarding gender, education, and age at diagnosis (Table 2). After the implementation of partnership care model, there was statistically significant difference between scores of mental health and subscale for symptoms of anxiety, depressive symptoms, social dysfunction, and physical complaints between the two groups (*P* < 0.05) (Table 3).

## 4. Discussion

In the present study, mental health scores in thalassemia patients were higher in both groups before intervention than the cut-off point that demonstrated poor mental health status of the patients. Naderi et al.'s study [25] shows that more than half (50.6%) of patients with thalassemia in southeastern Iran suffer from mental disorders. Other similar studies, in addition to the high prevalence of mental health, have reported high number of such disorders (such as anxiety and depression) requiring extensive follow-up [16, 26, 27]. Sadowski et al.'s study [28] shows that mental health problems in thalassemia patients (47.4%) is not only higher compared to healthy controls (26.3%), but also significantly higher than hemophilia patients (24.6%).

In our study, mean mental health score of thalassemia patient was much higher compared to other Iranian studies that have used the same tool [29, 30]. It seems that this difference in severity or incidence of different types of mental disorders in the reported studies may be due to the differences in the severity of the disorder, social environment, race, environmental support from patients, the treatments performed, and cultural view of coping with illness [27].

In our study, the subscales (symptoms of anxiety, depressive symptoms, social dysfunction, and physical complaints) in both groups before the intervention were higher than the specified cut-off point indicating impairment in these subscales. Naderi et al.'s study [25] shows in a study on thalassemia patients that 11.6% of patients had depressive symptoms, 8.5% had symptoms of anxiety, 7.3% had physical complaints, and 4.3% had social dysfunction that is consistent with our study. Salehi et al.'s study [30] shows that a high percentage of thalassemia patients were suspected to have or suffering from physical problems due to the impact of the disease on their appearance, early fatigue, anemia, or headache. Research has shown that thalassemia major can have a devastating impact on social activities of patients [1]; for example, Sadowski et al.'s study [28] shows that severe physical health problems in patients with thalassemia increase over time compared to healthy subjects, leading to social dysfunction and exacerbation of psychiatric disorders such as depression and anxiety. The results of multiple studies showed that the prevalence of depression and anxiety is significantly greater in patients with thalassemia than in healthy controls [12, 14, 15]. This anxiety may be due to fear of early death, concern in family formation, repeated blood transfusions, negative self-thoughts, and different feelings in these patients [15].

In our study, among the subscales, the highest score was related to depression. Research has shown relationship between thalassemia major and depression [26, 31]. Depression is confirmed as the most common psychiatric disorders in thalassemia patients in other studies [25, 28]. In the study of Marvasti et al.'s [32], the risk of depression was much higher in patients with thalassemia compared to healthy subjects. Keşkek et al.'s study [31] shows that not only is the prevalence of depression higher in patients with thalassemia compared to healthy subjects, but the severity of depression is also alarmingly higher in patients with thalassemia which needs urgent measures in these patients. Depression has negative and severe impacts on physical and mental health [33], which can be due to chronic nature of the disease, long treatment duration, early death expectation, the changed appearance, sense of deprivation, and social reflections, such as family, community, and school [12, 34].

Our study showed that the implementation of partnership care model is able to significantly improve the mental health of patients with thalassemia. Ratanasiripong et al.'s study [35] shows in a study that implementation of educational feedback program significantly decreased stress, anxiety, and depression, but the impact of Iranian partnership care model has not been studied. Alijany-Renany et al.'s study [19] shows that the quality of life of children with thalassemia significantly improved, compared to control group and before intervention and after implementation of partnership care model. Ghavidel et al.'s study shows that the implementation of partnership care model resulted in significant improvement in quality of life of hemodialysis patients in all aspects of physical, mental, and general health and life force and energy [36]. Partnership care model was also conducted on other patients. A significant and positive effect of the implementation of this model was approved on improving the quality of life of patients with hypertension [37], coronary arteries [38], chronic bronchiolitis [39], and stroke [40]. This model was implemented by Mamene et al., who concluded that this model will correct the lifestyle in various indexes and diet behavior [41]. Nayyeri et al.'s study [42] shows that the implementation of partnership care model is effective in increasing the quality of sleep in patients with heart failure. The results of all these studies, similar to our study, indicate the promising effect of the implementation of this model in different diseases.

Among the subscales, the results of our study showed that the implementation of this model could significantly improve the level of symptoms of anxiety, depressive symptoms, social dysfunction, and physical complaints in the intervention group compared to the control group. Research on this field has indicated that children with thalassemia, whose care-giver have less emphasis on providing information and explanations about the nature of the disease, suffer from more psychological distress that causes loneliness, depression, and anxiety and has negative impacts on emotional health, social functioning, and self-esteem of patients [7, 43]. Parviniannasab et al.'s study [44] shows that the implementation of this model significantly reduces the level of depression in thalassemia patients. The positive impact of this model on recovery and reduction of anxiety and depression in patients with heart failure [20], as well as patients with anxiety disorders [37], has been approved, which is consistent with our results.

Konstam et al.'s study [45] shows that educating the methods of controlling anxiety and cognitive therapy interventions can help special patients identify the physical and mental reasons of anxiety and depression and the ultimate response of these patients to education of the methods is decreased anxiety and depression.

## 5. Conclusion

The results of the present study indicated that patients with thalassemia major in Jiroft city are faced with severe mental health problems, especially depression, which requires planning of the authorities and using experienced consulters to educate life skills and how to face psychological and social problems to prevent and treat psychological disorders in these patients. Also, the findings of the present study indicated that partnership care model is effective and useful in improving the mental health of patients with thalassemia major; thus, regarding the efficacy of this model, besides its inexpensiveness and simplicity for families and children, implementation of such program is suggested for improvement of mental health of patients with thalassemia.

## Figures and Tables

**Table 1:** The order and content of the training sessions based partnership care model.

(1) Motivation	The purpose of this stage is to stimulate the patient. At this stage (the first meeting for 2 hours) a description of the study protocol was explained to the intervention group. Then training need was evaluated by assessing and identifying patients through history taking (purposive questions designed by thalassemia and clinical psychology specialists) and paraclinical examination that resulted in a list of problems in the field of treatment and care in three dimensions of unhealthy behavior, unawareness of diet, and inability to control psychological problems of the disease. The results and findings were discussed and exchanged with the active participation of team members including patients, clinical psychologists, nurses, and physician (according to areas of responsibility).

(2) Readiness	At this stage (the second session for one hour), the intervention group was informed about the nature of visits (training sessions and follow-up) and the objectives and duration of visits and they were provided with the timing of the training program.

(3) Involvement	This phase includes three visits to educational partnership and two visits to follow-up partnership. Three visits were scheduled in educational phase (each visit lasting 60 to 90 minutes with an interval of two weeks between appointments). The first visit was about the nature of the disease and treatment, the second visit was in the field of diet and activities, and the third visit was about psychological issues. In each visit, the contents were simply provided to patients by physician and nurses (according to areas of responsibility) and clinical psychologists (in the session of psychological issues) through lectures, PowerPoint presentations, pamphlets, and questions and answers. In the follow-up phase, two visits were scheduled (30–45 minutes for each visit with two-week interval). During these sessions, while discussing the problems of patients, the positive and negative results of education and previous measures were assessed and reviewed and necessary guidance was provided to correct errors.

(4) Evaluation	Phased evaluation was conducted in the beginning and end of each visit. In the final assessment (for 60–90 minutes), the GHQ-28 questionnaire was used to assess the impact of partnership care model on mental health of patients in the intervention group after three months. Then the same method of evaluation was conducted for the control group.

**Table 2:** The comparison of the variables between the experimental and control groups.

Variable groups	Intervention group *n* = 41	Control group *n* = 41	Statistical test and *P* value
	*N* (%)	*N* (%)	
Sex			
Male	21 (51.2)	23 (56.1)	Fisher *P* = 0.57
Female	20 (48.8)	18 (43.9)	
Education			
Primary school	29 (70.7)	30 (73.2)	*χ* ^2^ = 7.59 df = 4 *P* = 0.108
Secondary school	9 (22)	7 (17.1)	
High school	3 (7.3)	4 (9.8)	
Age at diagnosis			
<1 year	31 (75.6)	33 (80.5)	*χ* ^2^ = 9.15 df = 6 *P* = 0.165
1–3 years	3 (7.4)	2 (4.9)	
3–5 years	6 (14.6)	6 (14.6)	
>5 years	1 (2.4)	0 (0.0)	
History of other diseases			
Negative	31 (75.6)	33 (80.5)	*χ* ^2^ = 9.15 df = 6 *P* = 0.165
Diabetes	3 (7.4)	2 (4.9)	
Kidney disease	6 (14.6)	6 (14.6)	
Liver disease	1 (2.4)	0 (0.0)	
Family status			
Living with two parents	35 (85.4)	38 (92.7)	*χ* ^2^ = 2.24 df = 2 *P* = 0.326
Living with one parent	6 (14.6)	2 (4.9)	
Living without parents	0 (0.0)	1 (2.4)	

**Table 3:** Comparison of mental health and its subscales in the intervention and control groups before and after intervention.

Variable	Group	Intervention	Control	Independent *t*-test
		Mean ± SD	Mean ± SD	
Physical complaints	Before	6.2 ± 0.42	6.0 ± 0.73	*T* = 0.67, *P* = 0.50
	After	3.9 ± 0.56	5.8 ± 0.67	*T* = 1.21, *P* = 0.007
Paired *t*-test		*T* = 10.7, *P* = 0.000	*T* = 1.37, *P* = 0.18	

Anxiety	Before	8.4 ± 1.07	8.6 ± 1.17	*T* = 0.39, *P* = 0.86
	After	5.2 ± 1.54	9.0 ± 0.66	*T* = 7.1, *P* = 0.014
Paired *t*-test		*T* = 7.6, *P* = 0.000	*T* = 1.8, *P* = 0.104	

Social dysfunction	Before	9.6 ± 0.89	9.8 ± 0.37	*T* = 0.36, *P* = 0.76
	After	7.0 ± 1.73	9.2 ± 0.20	*T* = 2.15, *P* = 0.070
Paired *t*-test		*T* = 5.1, *P* = 0.007	*T* = 1.50, *P* = 0.21	

Depression	Before	10.8 ± 1.30	11.0 ± 0.70	*T* = 0.30, *P* = 0.11
	After	4.4 ± 1.14	11.2 ± 0.44	*T* = 5.21, *P* = 0.008
Paired *t*-test		*T* = 8.5, *P* = 0.001	*T* = 1.0, *P* = 0.37	

Total score of mental health	Before	35.2 ± 7.31	35.4 ± 7.83	*T* = 0.65, *P* = 0.35
	After	11.4 ± 3.06	35.2 ± 7.01	*T* = 8.1, *P* = 0.004
Paired *t*-test		*T* = 49.6, *P* = 0.000	*T* = 1.0, *P* = 0.39	
